# Sex Steroid Levels in Women With Hypopituitarism: A Case-controlled Observational Study

**DOI:** 10.1210/clinem/dgae197

**Published:** 2024-04-04

**Authors:** Catharina Olivius, Kerstin Landin-Wilhelmsen, Claes Ohlsson, Matti Poutanen, Penelope Trimpou, Daniel S Olsson, Gudmundur Johannsson, Åsa Tivesten

**Affiliations:** Wallenberg Laboratory for Cardiovascular and Metabolic Research, Department of Molecular and Clinical Medicine, Institute of Medicine, Sahlgrenska Academy, University of Gothenburg, S-413 45 Gothenburg, Sweden; Department of Medicine, Hospital of Halland, S-434 80 Kungsbacka, Sweden; Department of Internal Medicine and Clinical Nutrition, Institute of Medicine, Sahlgrenska Academy, University of Gothenburg, S-413 45 Gothenburg, Sweden; Department of Endocrinology, Sahlgrenska University Hospital, Region Västra Götaland, S-413 45 Gothenburg, Sweden; Department of Internal Medicine and Clinical Nutrition, Institute of Medicine, Sahlgrenska Academy, University of Gothenburg, S-413 45 Gothenburg, Sweden; Sahlgrenska Osteoporosis Centre, Centre for Bone and Arthritis Research at the Sahlgrenska Academy, University of Gothenburg, S-413 45 Gothenburg, Sweden; Department of Drug Treatment, Sahlgrenska University Hospital, Region Västra Götaland, S-413 45 Gothenburg, Sweden; Department of Internal Medicine and Clinical Nutrition, Institute of Medicine, Sahlgrenska Academy, University of Gothenburg, S-413 45 Gothenburg, Sweden; Sahlgrenska Osteoporosis Centre, Centre for Bone and Arthritis Research at the Sahlgrenska Academy, University of Gothenburg, S-413 45 Gothenburg, Sweden; Institute of Biomedicine, Research Centre for Integrative Physiology and Pharmacology, University of Turku, 205 20 Turku, Finland; Department of Internal Medicine and Clinical Nutrition, Institute of Medicine, Sahlgrenska Academy, University of Gothenburg, S-413 45 Gothenburg, Sweden; Department of Endocrinology, Sahlgrenska University Hospital, Region Västra Götaland, S-413 45 Gothenburg, Sweden; Department of Internal Medicine and Clinical Nutrition, Institute of Medicine, Sahlgrenska Academy, University of Gothenburg, S-413 45 Gothenburg, Sweden; Department of Endocrinology, Sahlgrenska University Hospital, Region Västra Götaland, S-413 45 Gothenburg, Sweden; Cardiovascular, Renal and Metabolism, BioPharmaceuticals R&D, AstraZeneca, S-431 83 Molndal, Sweden; Department of Internal Medicine and Clinical Nutrition, Institute of Medicine, Sahlgrenska Academy, University of Gothenburg, S-413 45 Gothenburg, Sweden; Department of Endocrinology, Sahlgrenska University Hospital, Region Västra Götaland, S-413 45 Gothenburg, Sweden; Wallenberg Laboratory for Cardiovascular and Metabolic Research, Department of Molecular and Clinical Medicine, Institute of Medicine, Sahlgrenska Academy, University of Gothenburg, S-413 45 Gothenburg, Sweden; Department of Endocrinology, Sahlgrenska University Hospital, Region Västra Götaland, S-413 45 Gothenburg, Sweden

**Keywords:** androgens, estrogens, women, hypopituitarism, hypogonadotropic hypogonadism, mass spectrometry

## Abstract

**Context:**

Women with hypopituitarism remain at increased risk of morbidity and mortality. Insufficient replacement of sex steroids has been suggested as a contributing factor, but sex steroid levels in women with hypopituitarism have not been comprehensively mapped.

**Objective:**

To quantify sex steroids in women with hypopituitarism by a high-sensitivity assay.

**Methods:**

Using a combination of clinical and biochemical criteria, women with hypopituitarism (n = 104) who started GH replacement in 1995 to 2014 at a single center were categorized as eugonadal or having hypogonadotropic hypogonadism (HH). A population-based cohort of women (n = 288) served as controls. Eugonadal women and controls were categorized as pre-/postmenopausal and HH women as younger/older (≤ or >52 years). Dehydroepiandrosterone (DHEA), androstenedione, testosterone, dihydrotestosterone, progesterone, 17αOH-progesterone, estradiol, and estrone were analyzed by a validated liquid chromatography-tandem mass spectrometry assay.

**Results:**

Among both premenopausal/younger and postmenopausal/older women, women with HH had lower levels of sex steroid precursors (DHEA, androstenedione) and androgens (testosterone and dihydrotestosterone) than controls. Progesterone, 17αOH-progesterone, estrone, and estradiol showed similar patterns. Women with HH and ACTH deficiency had markedly lower concentrations of all sex hormones than those without ACTH deficiency.

**Conclusion:**

This study demonstrates for the first time a broad and severe sex steroid deficiency in both younger and older women with HH, particularly in those with combined gonadotropin and ACTH deficiency. The health impact of low sex steroid levels in women with hypopituitarism requires further study, and women with combined gonadotropin and ACTH deficiency should be a prioritized group for intervention studies with sex hormone replacement.

Hypopituitarism is a rare condition in which there is a deficiency of 1, multiple, or all of the hormones produced by the anterior pituitary gland. Hypopituitarism has an estimated annual incidence of 4.21 cases per 100 000 and a prevalence of 45.5/100 000 that may be increasing (1). The mortality rate in men with hypopituitarism caused by benign nonfunctioning adenomas has decreased in recent decades and is now close to that of the background population (2, 3). By contrast, women with hypopituitarism remain at increased risk of mortality, as well as cardiovascular events and fractures (2-7). The pituitary gland produces hormones (the gonadotropins LH and FSH and ACTH) that control the production of sex hormones (estrogens, androgens, and progesterone), and insufficient replacement of sex steroids in women with hypopituitarism has been suggested as a factor contributing to this discrepancy between men and women (4, 7). Men with pituitary insufficiency and low androgen levels receive testosterone replacement whereas women with hypopituitarism and estrogen deficiency are only recommended estrogen replacement until the expected age of menopause and not androgen replacement (8-10). Lower androgen levels in women without any pituitary disorders have been reported to be associated with increased mortality (11), cardiovascular disease (12, 13), and lower bone mineral density (14, 15), but a comprehensive understanding of the physiological and pathophysiological roles of sex steroids, in particular androgens, in women is lacking.

Hypogonadotropic hypogonadism (HH), ie, deficiency of LH/FSH and thereby estrogen production, is diagnosed according to strict criteria combining clinical and laboratory variables in both pre- and postmenopausal women (9). By contrast, there is no established definition of androgen deficiency in women, and it is unclear how HH, alone or in combination with ACTH deficiency, affects the sex hormone profile (8). Progress in defining sex steroid deficiency, understanding its consequences, and optimizing its replacement therapy has been hampered by the lack of sensitive and specific analytical methods for determining sex hormone levels. The performance of immunoassays that are used in clinical routine is insufficient for measurements of the low levels of sex steroids (16-18) present in women, particularly after menopause, and in women with hypopituitarism, adrenal insufficiency, and ovarian failure. In such settings, the use of mass spectrometry-based analytical methods is required (19). High-quality sex steroid data from mass spectrometry in women with hypopituitarism may provide important clues to the regulation and importance of sex steroids in women, but studies to generate these data have not yet been performed.

The aim of this study was to determine the spectrum of sex steroid levels in a cohort of women with hypopituitarism, with and without HH and ACTH deficiency, using a validated (20) state-of-the-art liquid chromatography-tandem mass spectrometry (LC/MS-MS) assay, and to compare with a population-based sample of women.

## Methods

### Study Design and Participants

**Figure 1.**
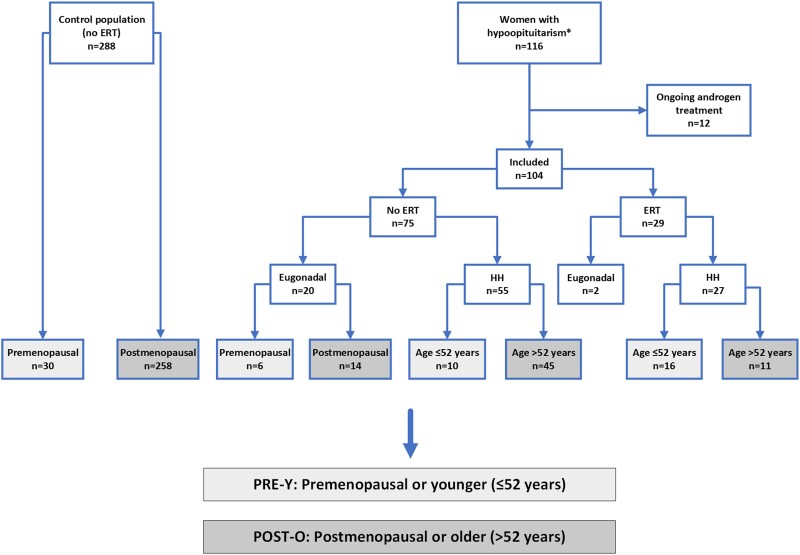
Flow-chart of the participating women. Light grey indicates PRE-Y women; dark grey indicates POST-O women. *Women with hypopituitarism who started GH treatment during the years 1995 to 2014 were evaluated for the present study. Two of the eugonadal patients were on birth control pills. Abbreviations: ERT, estrogen replacement therapy; HH, hypogonadotropic hypogonadism; PRE-Y, premenopausal or younger (≤52 years); POST-O, postmenopausal or older (>52 years).

This was a case-controlled observational study based on a registry of all adults who received replacement with GH at the Centre for Endocrinology and Metabolism, Sahlgrenska University Hospital (the Gothenburg Pituitary study) (21). Women with hypopituitarism who started GH replacement between 1995 and 2014 and had frozen blood samples available were evaluated for the present study, with the following exceptions: women with a diagnosis of hormonally active pituitary adenoma (except prolactinoma), extra-pituitary tumors or malignancies (except dermoid cysts and craniopharyngioma), serious systemic diseases or malformations. Among 116 evaluated women, 12 had ongoing androgen replacement at the time of the blood sampling and were excluded. After this exclusion, 104 women remained in this study (Fig. 1). The underlying diagnoses of the 104 women with hypopituitarism were nonfunctioning pituitary adenoma (n = 40), prolactinoma (n = 14), pituitary cyst (n = 11), hypopituitarism (n = 8), craniopharyngeoma (n = 7), empty sella (n = 6), Sheehan's syndrome (n = 6), hypophysitis (n = 5), idiopathic GH deficiency (n = 5), Kallman's syndrome (n = 1), and traumatic brain injury (n = 1).

The Gothenburg Pituitary study included examination (including anthropometry) by a physician and fasting blood collection at the start of GH replacement (defined as baseline) and after 6 months and 1 year and 2, 3, 5, 7, 10, 12, 17, 20, and 25 years of follow-up. At each examination, serum was collected and stored at −70 °C (21). Serum was collected on a random cycle day for the minor fraction of women who were cycling. For the present analysis of sex steroids, we selected the serum sample from the examination in 2008 or as soon after 2008 as possible but at least 3 years after baseline. Median (interquartile range) for the sample year was 2008 (2008-2011).

The control population consisted of women from the World Health Organization MONItoring of trends and determinants for CArdiovascular disease (WHO MONICA) study, who were examined in 2008. WHO MONICA was conducted in 38 countries and included a randomized population sample from the years 1985, 1990, and 1995 (22). In Gothenburg, 1200 women and 1200 men aged 25 to 64 years were examined at each time point. In 2008, there was a reexamination of the men and women who were included in 1995; 608 were invited, and 410 accepted, of whom 318 were women (23). The reexamination in 2008 included clinical examination (including anthropometry), questionnaire data (including medication use), and blood sampling. Serum was stored at −70 °C. In cycling women, blood samples were collected on cycle day 7 to 9. For the present study, 27 of the 318 women were excluded because they were on estrogen and/or progesterone at the time of the blood sampling, 2 were excluded because their serum was not found, and 1 woman was excluded due to known HH. The remaining 288 women from the 2008 reexamination in Gothenburg served as a control population in this study, none of whom were treated with androgens.

The studies were approved by the Regional Ethical Review Board in Gothenburg, Sweden. All participants provided written informed consent.

### Procedures

In the hypopituitary cohort, an endocrinology specialist (C.O.) made a retrospective evaluation of data in the medical records and in the registry. Hormone deficiencies, how they were diagnosed, and their replacement therapies were recorded. Deficiencies of ACTH, TSH, GH, and antidiuretic hormone were defined by the treating physician according to clinical guidelines. In the general cohort, all 104 women were GH deficient, 82 were LH/FSH deficient (had HH), 77 were TSH deficient, 55 were ACTH deficient, and 20 had diabetes insipidus. All women with ACTH deficiency received replacement therapy with corticosteroids, and all women with TSH, GH deficiency, and diabetes insipidus received replacement therapy with L-thyroxine, GH, and vasopressin, respectively. All women with prolactinoma (n = 14) had normal serum prolactin levels at the time of the blood sampling; 10 had previously undergone pituitary surgery, 1 had received pituitary radiation, and 3 were receiving treatment with cabergoline.

Retrospective evaluation of HH in the hypopituitary cohort was performed as previously described (24). The following stringent criteria, based on the recommendations in the Endocrine Society's Clinical Practice Guidelines (9), were applied (1): in premenopausal ages (≤52 years) (25): amenorrhea or severe oligomenorrhea without other gynecological explanation, supported by available measurements of serum estradiol, LH, FSH, and, in a few cases (n = 4), GHRH stimulation test (2); in postmenopausal ages (>52 years): amenorrhea with absence of both elevated FSH and LH and no estrogen replacement therapy (ERT) (3); in childhood-onset hypopituitarism: absence of puberty and puberty induction and (4) panhypopituitarism, where other assessment was impossible, eg, due to absence of FSH/LH measurements in postmenopausal women or ERT at the time of evaluation. For the present project, LH and FSH were reanalyzed to obtain LH, FSH, and sex steroid data from the same time point. The HH diagnosis was then reevaluated and was changed from the previous assessment (24) for 4 of the 104 women.

Among the eugonadal women with hypopituitarism and the women who served as controls, postmenopausal status was defined by elevated FSH and LH levels (FSH ≥27 IU/L; LH ≥5.2 IU/L). Women with lower than postmenopausal FSH levels were evaluated with respect to age, FSH, LH, and estradiol levels (performed by C.O.). As the menopausal status of the women with HH cannot be determined, HH women were categorized into age groups (52 years or younger; older than 52 years), according to the approximate mean age of menopause in the population (25).

Serum sex steroids [dehydroepiandrosterone (DHEA), progesterone, 17-alpha-hydroxyprogesterone (17αOH-progesterone), androstenedione, estrone, testosterone, dihydrotestosterone (DHT), and estradiol] were analyzed by an in-house validated LC/MS-MS assay, as described previously (20). The lower limit of quantification (LLOQ) for DHEA, progesterone, 17αOH-progesterone, androstenedione, estrone, testosterone, DHT, and estradiol was 250, 5, 20, 5, 0.5, 5, 13, and 0.5 pg/mL, respectively. SHBG was measured by a chemiluminescent-based SHBG assay validated for clinical use (LIAISON® SHBG, cat. no. 319020, DiaSorin; RRID: AB 2895155), and the DiaSorin LIAISON immunoanalyzer was used. Serum LH (cat. no. 07P9120) and FSH (cat. no. 07P4920) were analyzed by Chemiluminescent Microparticle Immunoassays, Abbott Laboratories, validated for clinical use.

### Statistical Analysis and Outcomes

The primary outcome variables were the levels of sex hormones across categories of women with hypopituitarism and control women. Sex steroid levels below LLOQ were set to LLOQ/√2 in analyses (26). The Shapiro–Wilk test and QQ-plots were used to determine normal distribution. Since most of the hormone data was not normally distributed, nonparametric tests were used. To compare hormone levels between groups, the Mann–Whitney U test and Kruskal–Wallis test with multiple pairwise comparisons by Dunn–Bonferroni test were used. Categorical variables were analyzed by Chi-square test or Fisher´s exact test. Quantile regression analysis was used to analyze the relationship between age and sex hormone levels in the control group. Percentiles were used to define 95% reference intervals. A *P-*value of < .05 was considered statistically significant. Statistical analyses were performed using the IBM SPSS (version 27; SPSS Institute) software.

## Results

### Characteristics of the Study Participants

Of the 104 women with hypopituitarism included in the study, 75 were not receiving ERT (Fig. 1) and were primarily studied. Eugonadal women with hypopituitarism and controls were categorized as premenopausal and postmenopausal, while women with HH were categorized as younger (≤52 years) or older (>52 years). Comparisons between women with hypopituitarism and controls were performed separately for premenopausal/younger women (PRE-Y) and postmenopausal/older women (POST-O, Fig. 1). The women with HH were further subcategorized by absence/presence of ACTH deficiency (Table 1). Only 5 eugonadal women with hypopituitarism had ACTH deficiency, which was determined to be too few for a separate analysis.

**Table 1.** Characteristics of the women with hypopituitarism and controls, without ERT

PRE-Y women					
	Premenopausal controls (n = 30)	PRE-Y women with hypopituitarism (n = 16)			
		Eugonadal (n = 6)	HH (n = 10)		
			All (n = 10)	Without ACTH deficiency (n = 3)	With ACTH deficiency (n = 7)
Age, years	45.0 (41.7-49.4)	41.0 (32.8-43.3)	48.0 (42.2-50.0)*^a^*	45.0 (34.0-47.5)	49.0 (46.0-50.0)
BMI, kg/m^2^	25.3 (22.4-27.4)	25.1 (21.8-27.5)	31.0 (25.7-41.8)*^a^*	45.0 (38.0-46.2)	28.7 (24.1-34.4)
TSH deficiency, n (%)	—	2 (33)	9 (90)*^d^*	2 (67)	7 (100)
Diabetes insipidus, n (%)	—	0 (0)	4 (40)	1 (33)	3 (43)
LH, IU/L	3.8 (2.4-7.4)	5.3 (3.6-8.1)	1.5 (0-2.4)*^b, e^*	2.2 (1.5-2.4)	0.4 (0-2.8)
FSH, IU/L	6.6 (24.6-11.5)	6.9 (6.1-7.9)	4.8 (0.3-6.6)	5.6 (4.8-5.9)	1.6 (0.3-7.2)
SHBG, nmol/L	77.8 (55.1-94.0)	59.5 (45.2-86.8)	48.2 (30.2-77.8)	50.0 (30.6-55.3)	46.5 (29.2-122)

POST-O women					
	Postmenopausal controls (n = 258)	POST-O women with hypopituitarism (n = 59)			
		Eugonadal (n = 14)	HH (n = 45)		
			All (n = 45)	Without ACTH deficiency (n = 21)	With ACTH deficiency (n = 24)
Age, years	66.0 (61.0-71.0)	58.5 (50.5-62.0)*^b^*	68.0 (62.0-72.0)*^f^*	68.0 (60.5-72.5)	68.0 (63.0-71.8)
BMI, kg/m^2^	26.0 (23.5-29.5)	29.0 (23.8-31.2)	28.6 (25.7-32.4)*^b^*	29.0 (26.3-33.3)	28.0 (25.2-31.6)
TSH deficiency, n (%)	—	6 (43)	35 (78)*^d^*	11 (52)	24 (100)*^h^*
Diabetes insipidus, n (%)	—	1 (7)	10 (22)	1 (5)	9 (38)^*c*^
LH, IU/L	22.1 (17.6-27.9)	16.0 (11.0-19.5)*^b^*	0.8 (0.2-3.7)*^c, e^*	3.7 (0.5-4.9)	0.4 (0.1-1.2)*^g^*
FSH, IU/L	62.2 (48.4-79.5)	43.0 (37.0-53.5)*^a^*	3.2 (1.5-13.5)*^c, e^*	13.0 (2.2-17.5)	2.4 (0.8-4.1)*^g^*
SHBG, nmol/L	79.0 (57.4-104)	38.7 (23.9-77.5)*^b^*	60.5 (39.3-85.3)*^a^*	61.0 (40.0-94.3)	59.0 (37.2-85.4)

Characteristics of the study participants at the time of blood sampling. Data are median (interquartile range) or n (%).

Kruskal–Wallis test, with multiple pairwise comparisons by Dunn–Bonferroni test, was used to compare the 3 main groups: controls, eugonadal, and HH-All. Categorical variables were analyzed by Fisher´s exact test. Mann–Whitney U-test was used to compare the 2 subgroups of HH: HH with and without ACTH deficiency.

Abbreviations: BMI, body mass index; ERT, estrogen replacement therapy; HH, hypogonadotropic hypogonadism; POST-O, postmenopausal or older (>52 years) women; PRE-Y, premenopausal or younger (≤52 years) women.

^*a*^ *P* < .05.

^*b*^ *P* < .01.

^*c*^ *P* < .001 vs controls.

^*d*^ *P* < .05.

^*e*^ *P* < .01.

^*f*^ *P* < .001 vs eugonadal.

^*g*^ *P* < .01.

^*h*^ *P* < .001 vs HH without ACTH deficiency.

The characteristics of the study participants who were not taking ERT at the time of the blood sampling, and divided into PRE-Y and POST-O groups, are shown in Table 1. The median age of PRE-Y and POST-O women was comparable between the controls and women with HH but lower in the eugonadal women with hypopituitarism. Body mass index was higher in the HH women compared to controls. As expected, gonadotropin levels differed across the groups and were lowest in HH women, particularly in those with ACTH deficiency.

### Levels of Sex Steroid Precursors, Androgens, and Progesterone in the Study Participants

**Figure 2.**
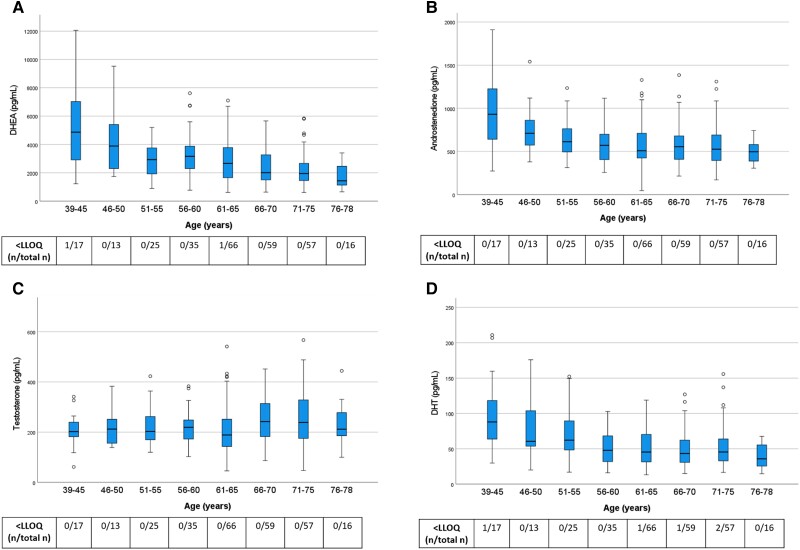
Androgen levels in control women from the general population across age groups. All women were without ERT. Box boundaries represent the 25th to 75th percentile, horizontal bars represent median values, whiskers represent lowest/highest values that is not an outlier, and circles represent statistical outliers. The levels were negatively correlated with higher age group for DHEA (*P* < .001), androstenedione (*P* < .01), and DHT (*P* < .001) but not for testosterone (*P* = .19; quantile regression analysis). Values < LLOQ were set to LLOQ/√2 in the analysis. Abbreviations: DHEA, dehydroepiandrosterone; DHT, dihydrotestosterone; ERT, estrogen replacement therapy; LLOQ, lower limit of quantification.

We first assessed serum levels of sex steroid precursors (DHEA, androstenedione) and active androgens (testosterone and DHT) across age categories in the control group (Fig. 2). With a few exceptions, androgen levels in the control group were above the assay LLOQ, confirming the high sensitivity of our assay. With increasing age, levels of DHEA, androstenedione, and DHT declined. By contrast, testosterone levels showed no statistically significant association with age (Fig. 2).

**Figure 3.**
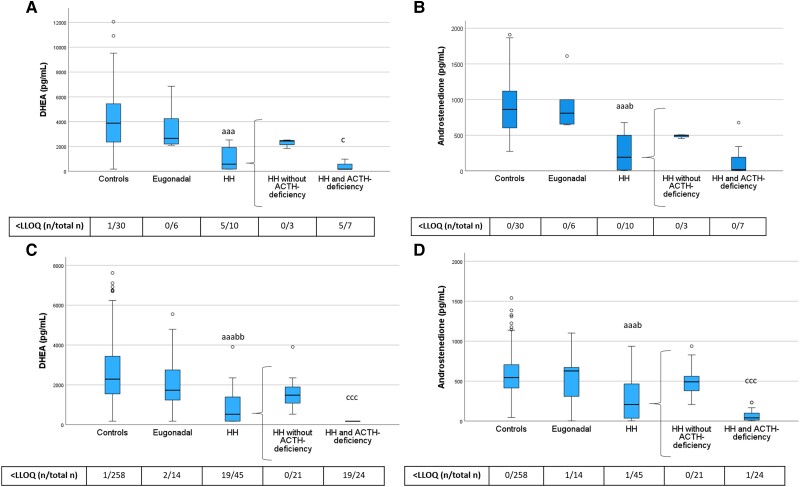
DHEA and androstenedione levels in PRE-Y (A, B) and POST-O (C, D) women with hypopituitarism and controls. All women were without ERT. Box boundaries represent the 25th to 75th percentile, horizontal bars represent median values, whiskers represent lowest/highest values that is not an outlier, and circles represent statistical outliers. ^aaa^*P* < .001 vs controls, ^b^*P* < .05, ^bb^*P* < .01 vs eugonadal, ^c^*P* < .05 ^ccc^*P* < .001 vs HH without ACTH deficiency. (Kruskal–Wallis test, with multiple pairwise comparisons by Dunn–Bonferroni test, was used to compare controls, engonadal, and HH. Mann–Whitney U-test was used to compare HH with and without ACTH deficiency.) Values < LLOQ were set to LLOQ/√2 in the analysis. Abbreviations: DHEA, dehydroepiandrosterone; ERT, estrogen replacement therapy; HH, hypogonadotropic hypogonadism; LLOQ, lower limit of quantification; PRE-Y, premenopausal or younger (≤52 years); POST-O, postmenopausal or older (>52 years).

**Figure 4.**
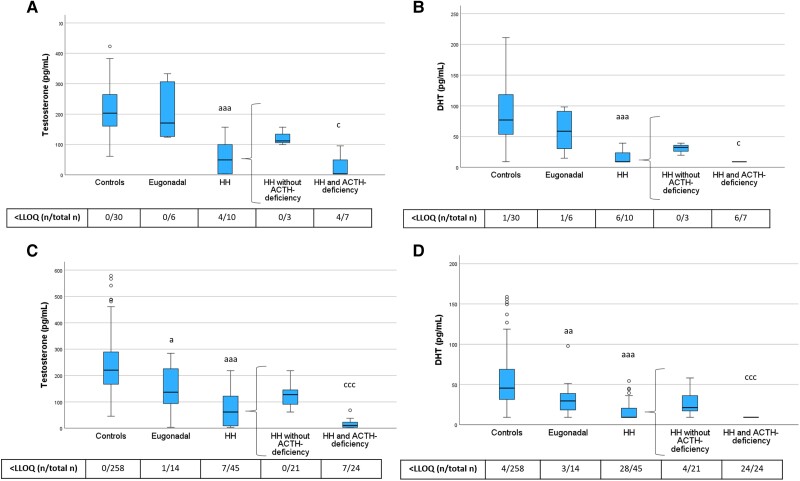
Testosterone and DHT levels in PRE-Y (A, B) and POST-O (C, D) women with hypopituitarism and controls. All women were without ERT. Box boundaries represent the 25th to 75th percentile, horizontal bars represent median values, whiskers represent lowest/highest values that is not an outlier, and circles represent statistical outliers. ^a^*P* < .05, ^aa^*P* < .01, ^aaa^*P* < .001 vs controls, ^c^*P* < .05, ^ccc^*P* < .001 vs HH without ACTH deficiency. (Kruskal–Wallis test, with multiple pairwise comparisons by Dunn–Bonferroni test, was used to compare controls, eugonadal and HH. Mann–Whitney U-test was used to compare HH with and without ACTH deficiency.) Values < LLOQ were set to LLOQ/√2 in the analysis. Abbreviations: ERT, estrogen replacement therapy; DHT, dihydrotestosterone; HH, hypogonadotropic hypogonadism; LLOQ, lower limit of quantification; PRE-Y, premenopausal or younger (≤52 years); POST-O, postmenopausal or older (>52 years).

**Figure 5.**
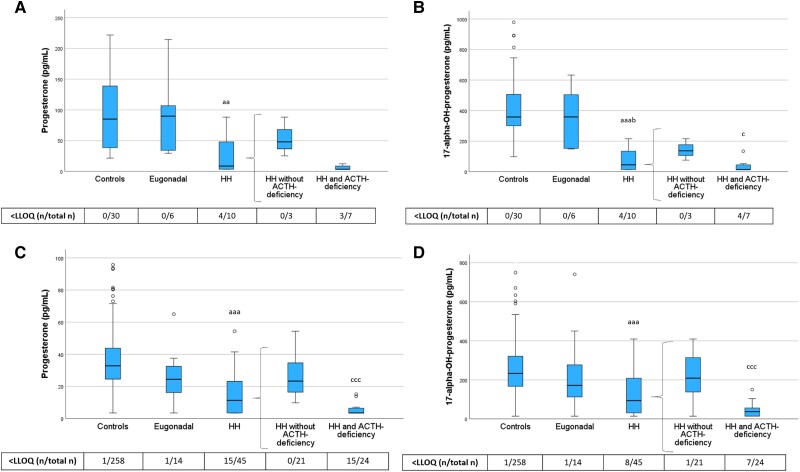
Progesterone and 17-alpha-OH-progesterone levels in PRE-Y (A, B) and POST-O (C, D) women with hypopituitarism and controls. All women were without ERT. Box boundaries represent the 25th to 75th percentile, horizontal bars represent median values, whiskers represent lowest/highest values that is not an outlier, and circles represent statistical outliers. ^aaa^*P* < .001, ^aa^*P* < .01 vs controls, ^b^*P* < .05 vs eugonadal, ^c^*P* < .05 ^ccc^*P* < .001 vs HH without ACTH deficiency. (Kruskal–Wallis test, with multiple pairwise comparisons by Dunn–Bonferroni test, was used to compare controls, eugonadal, and HH. Mann–Whitney U-test was used to compare HH with and without ACTH deficiency.) Values < LLOQ were set to LLOQ/√2 in the analysis. Abbreviations: ERT, estrogen replacement therapy; HH, hypogonadotropic hypogonadism; LLOQ, lower limit of quantification; PRE-Y, premenopausal or younger (≤52 years); POST-O, postmenopausal or older (>52 years).

Next, we examined serum levels of sex steroid precursors (Fig. 3), androgens (Fig. 4), and progesterone (Fig. 5) across the different groups of women with hypopituitarism who were not taking ERT and compared them to the control women, divided into PRE-Y and POST-O groups. In contrast to control women, a large fraction of the women with hypopituitarism had androgen values below the assay LLOQ, particularly for DHEA and DHT.

Levels of DHEA and androstenedione did not differ between eugonadal women with hypopituitarism and controls but were lower in HH women compared with both controls and eugonadal women (Fig. 3). HH women with ACTH deficiency had markedly lower levels of DHEA than those without ACTH deficiency, and this group also showed the highest frequency of DHEA levels below the assay LLOQ. Androstenedione showed a similar pattern as DHEA.

Levels of testosterone and DHT showed a similar pattern as the sex steroid precursors (Fig. 4). Testosterone and DHT levels were lower in HH women compared with controls, and HH women with ACTH deficiency had the lowest testosterone and DHT levels. In POST-O women, levels of testosterone and DHT were lower in eugonadal women with hypopituitarism compared to controls (Fig. 4). To address the potential role of ACTH deficiency for this observation, we performed a sensitivity analysis in which we excluded POST-O eugonadal women with hypopituitarism and ACTH deficiency (n = 4). After this exclusion, levels of testosterone (median 154, interquartile range 99.2-237 pg/mL) and DHT (32.4, 20.2-46.2 pg/mL) in POST-O eugonadal women with hypopituitarism (n = 10) were not different from those in postmenopausal control women (*P* = .34 and *P* = .26 by Mann–Whitney U test, respectively).

We also showed that levels of progesterone and its downstream metabolite 17α-hydroxyprogesterone were reduced in HH women but not in eugonadal women with hypopituitarism (Fig. 5). Further, both hormones were lowest in HH women with ACTH deficiency.

As described previously, levels of sex steroid precursors, androgens, and progesterone in HH women without ACTH deficiency were generally higher than in those with ACTH deficiency (Figs. 3-5). In further exploratory analyses (Mann–Whitney U test) among POST-O women without ERT, we directly compared levels of sex steroid precursors, androgens, and progesterone in HH women without ACTH deficiency with the levels in controls. This analysis showed that DHEA (*P* < .001), testosterone (*P* < .001), DHT (*P* < .001), and progesterone (*P* < .01) were lower in this group than in controls, while androstenedione (*P* = .11) and 17α-hydroxyprogesterone (*P* = .41) levels were not.

Androgen/sex steroid precursor levels in HH women were also compared in women who were taking or not taking ERT (see Fig. 1; characteristics in Supplementary Table S1) (27) and further subcategorized by ACTH deficiency. Overall, no striking differences in DHEA, androstenedione, testosterone, or DHT levels were found between HH women irrespective of ERT intake (Supplementary Figs. S1 and S2) (27).

### Levels of Estradiol and Estrone in the Study Participants

**Figure 6.**
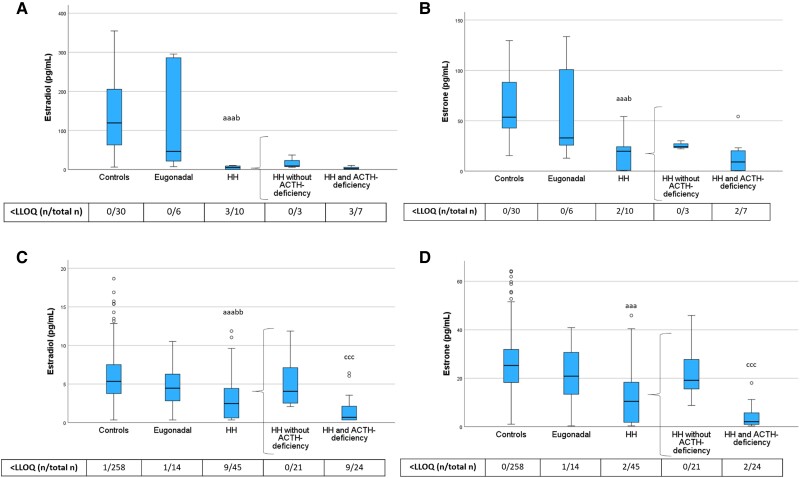
Estradiol and estrone levels in PRE-Y (A, B) and POST-O (C, D) women with hypopituitarism and controls. All women were without ERT. Box boundaries represent the 25th to 75th percentile, horizontal bars represent median values, whiskers represent lowest/highest values that is not an outlier, and circles represent statistical outliers. ^aaa^*P* < .001 vs controls. ^b^*P* < .05, ^bb^*P* < .01 vs eugonadal. ^ccc^*P* < .001 vs HH without ACTH deficiency. (Kruskal–Wallis test, with multiple pairwise comparisons by Dunn–Bonferroni test, was used to compare controls, eugonadal, and HH. Mann–Whitney U-test was used to compare HH with and without ACTH deficiency.) Values < LLOQ were set to LLOQ/√2 in the analysis. Abbreviations: ERT, estrogen replacement therapy; HH, hypogonadotropic hypogonadism; LLOQ, lower limit of quantification; PRE-Y, premenopausal or younger (≤52 years); POST-O, postmenopausal or older (>52 years).

In the PRE-Y category, HH women showed lower estradiol levels than both control women and eugonadal women with hypopituitarism (Fig. 6), confirming the diagnosis of HH. Interestingly, the same was true also among POST-O women. Further, among POST-O women, estradiol levels were strikingly lower in HH women with ACTH deficiency than those without. Estrone followed a similar pattern.

### Sex Hormone Levels Below the Reference Limits in POST-O Women With Hypopituitarism

We first calculated 95% reference ranges for sex steroids in postmenopausal control women and then assessed the fraction of POST-O women with hypopituitarism showing sex steroid levels below the lower reference ranges (Table 2). Only a minor fraction of the eugonadal women with hypopituitarism had low sex steroid levels. By contrast, a large fraction of the HH women had DHEA, androstenedione, testosterone, DHT, progesterone, and estrone levels below the reference levels. Of the HH women with ACTH deficiency, all had androgen/sex steroid precursor levels below the reference ranges, and the majority had below-reference levels for progesterone, estrone, and estradiol (83%, 88%, and 63%, respectively).

**Table 2.** Fraction of POST-O women with hypopituitarism with sex hormone levels below lower reference limit

	95% reference interval in postmenopausal control women (n = 258), pg/mL	POST-O women with hypopituitarism with sex hormone levels below lower reference limit, n (%)			
		Eugonadal (n = 14)	HH (n = 45)		
			All (n = 45)	Without ACTH deficiency (n = 21)	With ACTH deficiency (n = 24)
DHEA (pg/mL)	665-6522	2 (14)	25 (56)*^b^*	1 (5)	24 (100)*^c^*
Androstenedione (pg/mL)	240-1161	2 (14)	25 (56)*^b^*	1 (5)	24 (100)*^c^*
Testosterone (pg/mL)	93.5-516	3 (21)	30 (67)*^b^*	6 (29)	24 (100)*^c^*
DHT (pg/mL)	16.9-124	3 (21)	29 (64)*^b^*	5 (24)	24 (100)*^c^*
Progesterone (pg/mL)	13.1-125	1 (7)	23 (51)*^b^*	3 (14)	20 (83)*^b^*
17αOH-progesterone (pg/mL)	92.1-899	3 (21)	22 (49)	2 (10)	20 (83)*^c^*
Estradiol (pg/mL)	1.4-46.3	1 (7)	15 (33)	0 (0)	15 (63)*^c^*
Estrone (pg/mL)	9.2-86.1	2 (14)	22 (49)*^a^*	1 (5)	21 (88)*^c^*

All women were without ERT.

DHEA, androstenedione, testosterone, and DHT may be converted to nmol/L by multiplying given values by 0.00347, 0.00349, 0.00347, and 0.00345, respectively. Progesterone and 17αOH-progesterone may be converted to nmol/L by multiplying given values by 0.00318 and 0.00303, respectively. Estradiol and estrone may be converted to pmol/L by multiplying given values by 3.67 and 3.70.

Abbreviations: DHEA, dehydroepiandrosterone; DHT, dihydrotestosterone; ERT, estrogen replacement therapy; HH, hypogonadotropic hypogonadism; POST-O, postmenopausal or older (>52 years) women.

^*a*^ *P* < .05.

^*b*^ *P* < .01 vs eugonadal.

^*c*^ *P* < .001 vs HH without ACTH deficiency (Chi-square test or Fisher's exact test).

## Discussion

Insufficient replacement of sex steroids has been suggested as a factor contributing to increased risk of morbidity and mortality in women with hypopituitarism (4, 7). However, sex steroid levels in women with hypopituitarism have not been comprehensively mapped. In this case-controlled observational study of women with well-defined hypopituitarism and a population-based cohort of women as controls, we assessed sex hormone levels by using a validated, high-sensitivity LC/MS-MS method. Irrespective of age, women with HH had lower levels of sex steroid precursors (DHEA, androstenedione) and androgens (testosterone and dihydrotestosterone) than controls, while there were fewer alterations in eugonadal women with hypopituitarism. Similar patterns were observed for progesterone, 17αOH-progesterone, estrone, and estradiol. Women with HH and ACTH deficiency had markedly lower levels of all sex hormones than those without ACTH deficiency.

This is the first study presenting comprehensive sex steroid profiles measured by a highly sensitive mass spectrometry assay in women with varying severity of hypopituitarism. A study using column chromatography-preceded radioimmunoassay reported that mean serum DHEA sulphate, testosterone, and androstenedione levels were lower in women with both HH and ACTH deficiency compared with HH only (28), in line with our findings. Here we also report that DHEA, DHT, progesterone, 17αOH-progesterone, estrone, and estradiol levels were very low in this patient group. In addition, we addressed sex steroid levels in younger and older women separately, demonstrating for the first time a broad and severe sex steroid deficiency in both younger and older women with HH and ACTH deficiency. By contrast, in women with HH without ACTH deficiency, most sex steroid levels were within the eugonadal range. Thus, ACTH deficiency seems to be the main factor associated with low sex steroid levels in women with hypopituitarism.

Testosterone and its metabolite DHT are produced by the ovaries and the adrenals as well as via peripheral conversion of sex steroid precursors (29, 30). DHEA and androstenedione are mainly of adrenal origin but are also produced by the ovaries (30-32). In women with both gonadotropin and ACTH deficiency, sex steroid production from both the adrenals and the ovaries is compromised. These women would therefore be expected to display more severe androgen/sex steroid precursor deficiency than women with, for example, primary adrenal insufficiency (Addison's disease) or primary ovarian failure (POI). An earlier study using LC-MS/MS reported that median testosterone levels in pre- and postmenopausal women with Addison´s disease were 126 and 43 pg/mL, respectively (33). Another study using LC/MS-MS reported a median testosterone level of 202 pg/mL in younger women with POI (34). We found that median testosterone levels were 112/128 pg/mL in younger/older HH women without ACTH deficiency and <5/10 pg/mL in younger/older HH women with ACTH deficiency. Taken together, these results indicate that testosterone levels are indeed lower in hypopituitary women with combined gonadotropin and ACTH deficiency than in women with Addison's disease or POI.

As our study is observational, we cannot evaluate the role of ACTH deficiency per se vs that of glucocorticoid replacement or other confounders. Glucocorticoids are known to decrease androgen levels via inhibitory actions at multiple levels, including the pituitary (29). It is also conceivable that those with ACTH deficiency have a more severe HH. Of note, women with Addison's disease also require glucocorticoid replacement but yet appear to have higher serum testosterone levels than women with combined HH and ACTH deficiency, as discussed earlier. Irrespective of the underlying mechanism, the results indicate that hypopituitary women with both HH and ACTH deficiency have markedly reduced androgen levels, while HH women without ACTH deficiency have relatively less impact on the androgen levels.

In the present study, the levels of progesterone and its downstream metabolite 17αOH-progesterone followed the pattern of other sex steroids in both younger and older women with hypopituitarism. Progesterone may be of both ovarian and adrenal origin and is a well-studied reproductive hormone in premenopausal women. Preclinical studies indicate that progesterone may act as a precursor to active androgens (35), but levels and possible roles of progesterone in men and postmenopausal women remain unclear, and there are few cohorts in which serum progesterone levels have been analyzed by high-performance methodology (36).

This study showed, as expected, low estradiol and estrone levels among younger HH women compared with premenopausal controls. Somewhat unexpectedly, the older HH women also had lower estradiol and estrone levels than the postmenopausal controls, especially those with combined HH and ACTH deficiency. This finding is in line with a recent study showing that estradiol (measured by gas chromatography-tandem mass spectrometry) is reduced by ovariectomy in postmenopausal women, suggesting continued estradiol production by the ovaries after menopause (31). There may also be a significant peripheral production of estrogens from sex steroid precursors/androgens, which continues after menopause but is lost in women with ACTH deficiency. This finding further emphasizes the broad and severe sex steroid deficiency independently of age in HH women with concomitant ACTH deficiency.

Among women with HH, we could not detect any differences in androgen levels between those taking or not taking ERT. A previous study detected no major differences in androgen levels in women with hypopituitarism with and without ERT (28), in line with our findings. This lack of difference is surprising given that ERT has been shown to suppress endogenous androgen secretion (37, 38). However, one limitation is that the present groups were small and the women with ERT were somewhat younger than those without, which potentially may obscure any differences.

An age-related decline of the levels of DHEA, androstenedione, and DHT but not testosterone was detected in the control women in our study. In 2 previous studies using LC/MS-MS, the age-related decline of testosterone was less pronounced than that of other androgens (36, 37). The 95% reference intervals for androgens in postmenopausal women in these (36, 37) and other studies (34, 39) were comparable to those of the present study. DHT presents greater analytical challenges, and relatively few studies have addressed DHT levels by LC-MS/MS in women. An upper reference limit of serum DHT in postmenopausal women (0.462 nmol/L corresponding to 133 pg/mL) similar to ours (124 pg/mL) has previously been reported (36), but no previous study has reported a lower reference limit for DHT in postmenopausal women (here 16.9 pg/mL). An early study reported that DHT levels were decreased by more than half in post- vs premenopausal women, with a mean of 30 pg/mL in postmenopausal women (40), again in accordance with our data.

To date, ERT is recommended to younger, but not older, women with estrogen deficiency due to HH (9). In contrast to men, women with low androgen levels are not recommended testosterone replacement, and there is no established definition of androgen deficiency in women (8, 10). DHEA is not routinely replaced in either men or women. There are, however, ample data suggesting important roles of sex steroids in women (8, 12, 13, 32, 41-47). The fact that women with hypopituitarism remain at increased risk of mortality, as well as of cardiovascular events and fractures (2-7), emphasizes the need for further research on the role of sex steroids and sex steroid replacement. Our findings suggest that HH women with concomitant ACTH deficiency should be a prioritized group for intervention studies with sex hormone replacement.

A major limitation of the study is the observational study design, with an inherent high risk of bias and confounding. Further, some of the study groups were small, and the premenopausal control women were too few for determination of reference intervals. Another limitation is that blood samples from premenopausal controls were collected on cycle day 7 to 9, while blood samples from the few younger eugonadal women with hypopituitarism were collected on a random cycle day, which may affect the comparison between controls and eugonadal women with hypopituitarism. The heterogeneity of underlying diagnoses and degree of hypopituitarism may be considered as a limitation. However, the aim was to include all women with hypopituitarism during the prespecified time period, even those with isolated GH deficiency, to examine sex steroid levels across a wide spectrum of hypopituitarism and underlying causes. Another reason to include all women with hypopituitarism was to increase the sample size to increase power and also not to have subjective exclusions of cases. All patients with prolactinoma were well controlled with normal serum prolactin levels. Since low estrogen levels result in decreased SHBG synthesis, which alters the levels of free/bioavailable androgens, differences in androgen levels reported in this study may not reflect changes in free or bioavailable fractions.

The major strength of the study is that sex hormone levels in both cases and controls were measured simultaneously by a validated and highly sensitive assay, providing unique data of sex hormone levels in women with hypopituitarism and a population-based control group. Another strength is that all consecutive women with hypopituitarism and GH replacement from our center from 1995 until 2014 were evaluated for the study, as well as a large number of control women from the general population.

In conclusion, this study demonstrates for the first time a broad and severe sex steroid deficiency in both younger and older women with HH, particularly in those with combined gonadotropin and ACTH deficiency. The health impact of low sex steroid levels in women with hypopituitarism requires further study, and women with combined gonadotropin and ACTH deficiency should be a prioritized group for intervention studies with sex hormone replacement.

## Data Availability

Considering issues of patient confidentiality and restrictions in institutional review board permissions, original deidentified data is only available through specific reasonable request to the corresponding author and material transfer agreement following EU regulations.
